# Mycobacterial glycolipids di-*O*-acylated trehalose and tri-*O*-acylated trehalose downregulate inducible nitric oxide synthase and nitric oxide production in macrophages

**DOI:** 10.1186/s12865-015-0102-3

**Published:** 2015-06-23

**Authors:** Patricia Espinosa-Cueto, Marina Escalera-Zamudio, Alejandro Magallanes-Puebla, Luz María López-Marín, Erika Segura-Salinas, Raúl Mancilla

**Affiliations:** Departamento de Inmunología, Instituto de Investigaciones Biomédicas, Universidad Nacional Autónoma de México, Mexico City, Mexico; Institute of Zoo and Wildlife Research, Leibniz, Germany; Centro de Física Aplicada y Tecnología Avanzada, Universidad Nacional Autónoma de México, S ⁄ N, Ciudad Universitaria, 04510 Mexico City, Mexico

**Keywords:** Tuberculosis, Glycolipids, Nitric oxide, iNOS

## Abstract

**Background:**

Tuberculosis (TB) remains a serious human health problem that affects millions of people in the world. Understanding the biology of *Mycobacterium tuberculosis* (Mtb) is essential for tackling this devastating disease. Mtb possesses a very complex cell envelope containing a variety of lipid components that participate in the establishment of the infection. We have previously demonstrated that di-*O*-acylated trehalose (DAT), a non-covalently linked cell wall glycolipid, inhibits the proliferation of T lymphocytes and the production of cytokines.

**Results:**

In this work we show that DAT and the closely related tri-*O*-acylated trehalose (TAT) inhibits nitric oxide (NO) production and the inducible nitric oxide synthase (iNOS) expression in macrophages (MØ).

**Conclusions:**

These findings show that DAT and TAT are cell-wall located virulence factors that downregulate an important effector of the immune response against mycobacteria.

## Background

Tuberculosis (TB) remains a serious health problem, with 8.8 million new cases and 1.4 million deaths reported in 2013 [1]. An understanding of disease pathogenesis could lead to a rational strategy to fight TB. Along with the prolonged coevolution with man, the bacillus has developed the ability to neutralize the macrophages (MØ), which are usually very efficient to kill intracellular microbes. The adaptive capacity of mycobacteria resides mainly in the cell wall, a structure of high complexity composed of a covalently linked arabinogalactan-peptidoglycan backbone with covalently attached mycolic acids, as well as abundant non-covalently linked lipid components [2]. It is known that several of these lipids are capable of blocking the anti-mycobacterial host responses. Indeed, virulence of Mtb isolates has been associated with the cell wall lipid components. For instance, the high pathogenicity of W/Beijing isolates seems to be related to cell wall lipids that upregulate a TH2 immune response that favors the infection [3]. Once delipidated, the mycobacteria lose the ability to block the fusion of the phagosome with the lysosome, and total extractable lipids inhibit T-cell and MØ functions [4]. Peptidoglycan inhibits the macrophage response to IFN-γ at a transcriptional level [5]. Among the cell wall glycolipids, excels the effects on immune response caused by trehalose-6,6-dymicolate (cord factor), which promotes pro-inflammatory cytokine production, influences the persistence of mycobacteria within MØ and retards phagosome maturation [6]. Also important is the lipoarabinomannan, as it regulates phagosome maturation and inhibits acquired immunity [7]. We have shown previously that di-*O*-acylated trehalose (DAT), a non-covalently attached mycobacterial cell wall glycolipid, downregulates the proliferation of T cells and the production of cytokines, two essential features of adaptive immunity [8]. In this work, we show that DAT and its more heavily lipidated homologue, tri-*O*-acylated trehalose (TAT), downregulates the inducible nitric oxide synthase (iNOS) expression and nitric oxide (NO) production in MØs, which are effector components that are important in the immune response against mycobacteria.

## Results

### Isolation of DAT and TAT from *mycobacterium fortuitum*

**Fig. 1:**
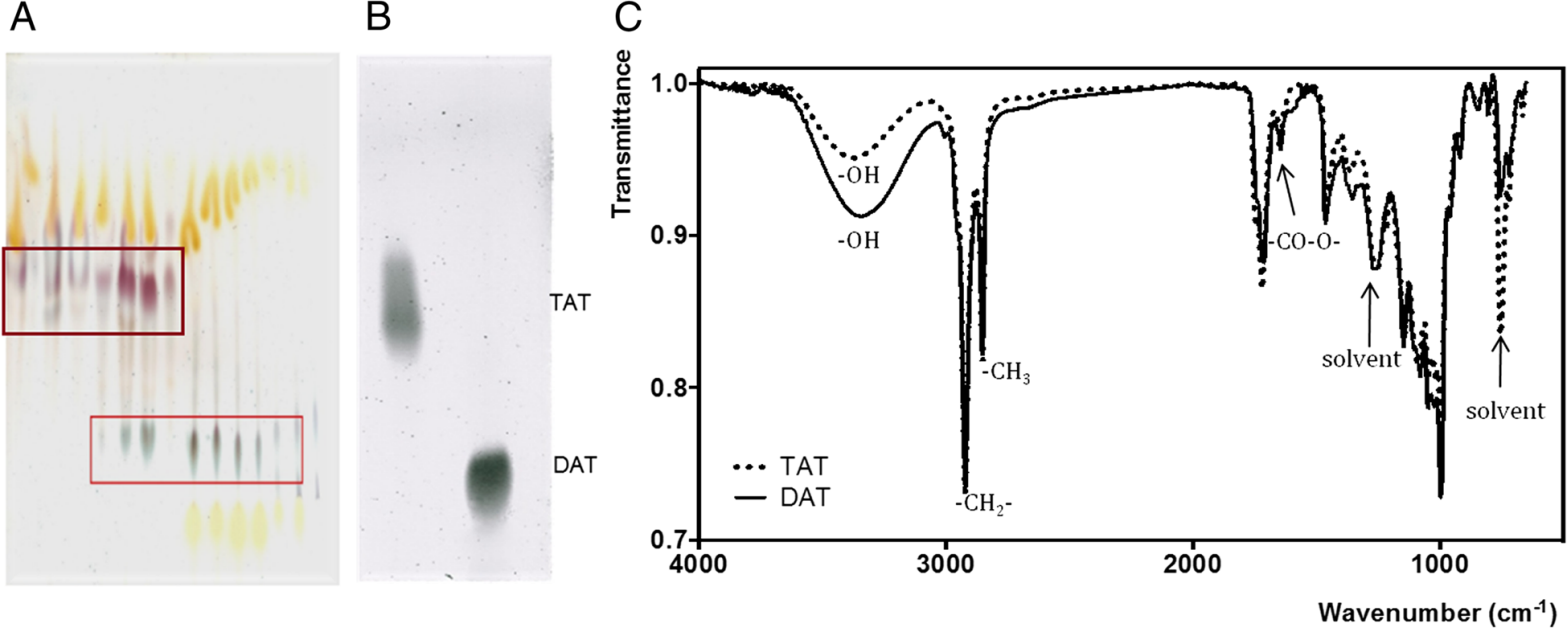
Purification and chemical characterization of DAT and TAT. Thin-layer chromatography of *M. fortuitum* ATCC 6841 unfractionated lipids showing TAT and DAT locations (**a**). Thin-layer chromatography of isolated TAT and DAT (**b**). Fourier Transform Infra-Red spectroscopy of DAT (solid line) and TAT (dotted line) (**c**)

*M. fortuitum* has been used as an alternative source of acyl trehaloses, which belong to a lipid family featuring virulent Mtb strains [9]. Non-mycoloyl fatty acylated trehaloses occur in virulent strains of the Mtb complex but are either absent or minimally represented in avirulent members of the complex, such as the H37Ra Mtb isolate or the vaccine strains *M. bovis* BCG and SO2 [9]. However, DAT and TAT have been described in *M. fortuitum* [10] and, taken from this source, they have been found to mimic both antigenicity and immunoregulation activities of the Mtb native compounds [8, 11]. Thin-layer-chromatography analyzes of crude lipid extracts are shown in Fig. 1. Acid/anthrone reagent was used to display sugar-containing lipids, which developed as blue spots, allowing the identification of two medium-polarity glycosyl-containing lipids in *M. fortuitum* (Fig. 1a). According to their mobility in chloroform-methanol (80:20, vol/vol) onto the silica gel layer, glycolipids with Rf values of 0.37 and 0.64-0.68 were tentatively identified as DAT and TAT, respectively (Fig. 1b). After their purification by column chromatography on Florisil and silica-gel solid-phase extraction, the isolated glycolipids were further characterized by Fourier Transform Infra-Red (FTIR) spectroscopy, (Fig. 1c). Both spectra share characteristic bands of glycosylated lipids, as expected. The characteristic band at 3320 and 3350 cm^−1^ indicates the presence of an oxygen-hydrogen bond in hydroxyl groups(-OH) in the sugar moieties of DAT y TAT, respectively. Absorption bands at 2922 cm^−1^ and 2853 cm^−1^ were assigned to the symmetric stretch of methylene (-CH_2_-) and methyl (-CH_3_) groups of aliphatic chains, respectively. The peak located at 1647 cm^−1^ indicates the presence of carboxyl ester groups (-CO-O-). Finally, the characteristic FTIR fingerprint of mycobacterial acylated trehaloses is shown in the region from about 1500 to 500 cm^−1^, due to all manner of bending vibrations of these molecules, the so-called mycosides F [10]. The concentration of endotoxin was measured by the Lymulus assay test and was undetectable.

### DAT and TAT inhibit no production by Murine MØs

**Fig. 2:**
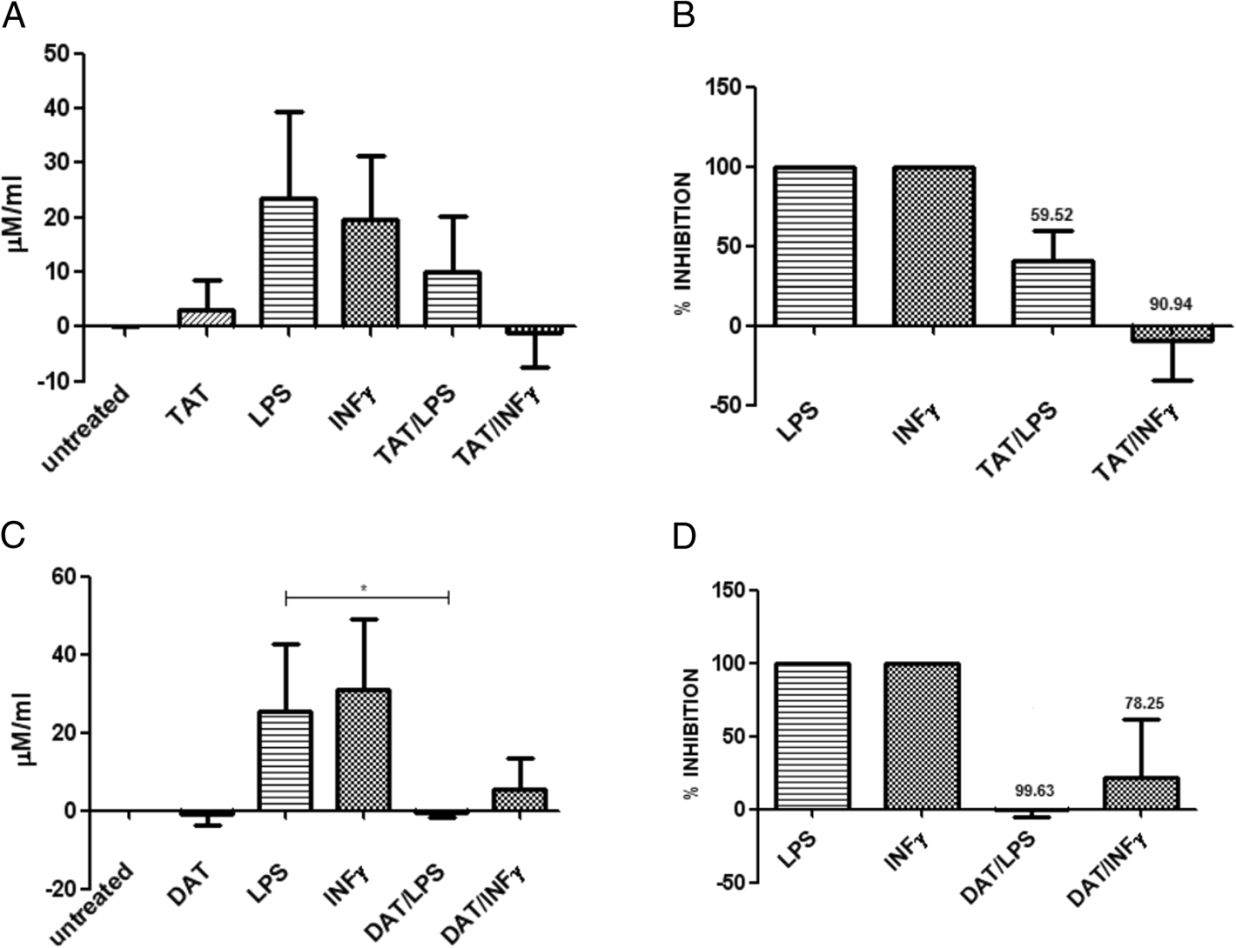
DAT and TAT downregulate NO production induced by LPS and IFN-γ in bone marrow-derived MØs. The isolated TAT or DAT were dissolved in hexane/methanol and placed in the wells. After solvent evaporation 1 × 10^6^, MØs were added to the wells. Afterward LPS (500ng) or IFN-γ (250ng) were added to the cells. After 24 h, the culture medium was collected, and NO was measured by the Griess reaction (**a, c**). The percent inhibition of NO production induced by TAT (**b**) and DAT (**d**) are shown. Results of four experiments are presented. Data regarding LPS against DAT/LPS was analyzed using an unpaired t-test with Welch’s correction to assess the statistical significance *p < 0.05

To analyze the effects of DAT and TAT on NO production in bone marrow-derived MØs, we first established the optimal conditions to carry out the assay. The MØs activated with 500ng of LPS released 23.4 μM/ml of NO in 24 h. When 20 ng of TAT were added to the cells, NO production was reduced by 59.52 % (Fig. 2a, b). Interestingly, the inhibitory effects of TAT on NO production induced by IFN-γ were higher (up to 90.9 %) (Fig. 2a, b). As for the effects of DAT, the inhibition of the production of NO induced by LPS was almost total and up to 78.2 % in cells activated with IFN-γ (Fig. 2c, d).

### DAT and TAT inhibit the inos expression in Murine MØ

**Fig. 3:**
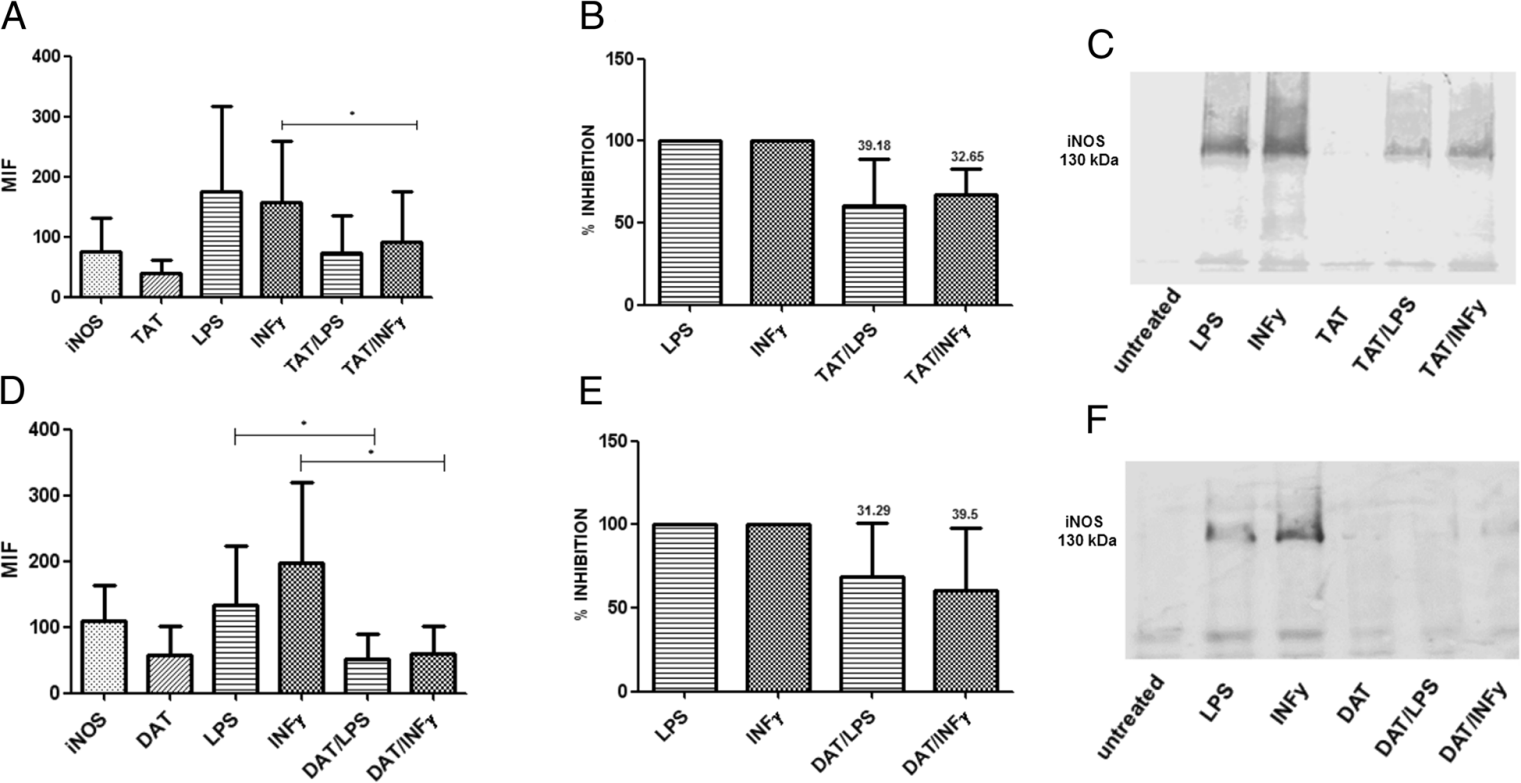
DAT and TAT downregulate iNOS expression. MØs were treated with the glycolipids and activated with LPS or IFN-gamma as described for Fig. 2. The expression of iNOS was analyzed by flow cytometry with permeabilized cells (**a**,**d**). The percent inhibition of iNOS is shown (**b**,**e**). After 24 h, the MØs were obtained, lysed and the proteins were electrophoresed and transferred to a PVDF membrane for Western blot with a monoclonal antibody to iNOS (**c**,**f**). Results of four experiments are shown. Data regarding LPS against DAT/LPS and INF-γ against DAT/IFN- γ, were analyzed with a paired t-test to assess the statistical significance *p < 0.05

In view of the involvement of iNOS in the regulation of NO production, we studied the effects of DAT and TAT on iNOS expression induced either by LPS or IFN-γ in bone marrow-derived MØs (Fig. 3). These assays showed that TAT reduced up to 39.18 % the expression of iNOS in cells activated with LPS. Similarly, the expression of iNOS in cells activated with IFN-γ was downregulated by TAT in 32.6 % (Fig. 3a, b). DAT also inhibited the expression of iNOS in 31.2 % after activation with LPS and 39.5 % when the cells were stimulated with IFN-γ (Fig. 3d, e). Western blot analyzes also showed a decreased expression of iNOS after exposure of MØs to acyl-trehaloses. However, this technique indicated a more pronounced effect on the di-*O*-acylated molecule (Fig. 3c, f).

## Discussion

NO is a bioactive gas produced by MØs activated with INF-γ through the catalytic action of iNOS [12]. The production of NO is an important host defense mechanism in Mtb infection. Mice with disrupted iNOS genes are highly susceptible to TB and develop progressive disease [12]. In murine models of tuberculosis, NO is known to enhance the phagosomal maturation, thus promoting macrophage killing of the bacilli [12, 13]. The relevance of NO for controlling the bacilli in humans remains controversial. However, various data support that the NO/iNOS system plays a role in tuberculosis disease. For instance, Mtb triggers the production of NO and iNOS in MØs from both healthy and tuberculous individuals [14, 15]. Patients with active pulmonary tuberculosis exhale NO, which is associated with increased production of iNOS by alveolar macrophages [16]. Moreover, histopathologic studies have shown that iNOS is expressed in human tuberculous granulomas [17]. Recently, our group demonstrated the expression of iNOS in MØs and multinucleated Langhans-type giant cells, as well as extensive MØs nitrosylation within bovine tuberculosis granulomas [18]. Finally, some iNOS polymorphisms seem to be associated with an increased susceptibility to TB in humans [19].

At high concentration, NO kills Mtb very efficiently in vitro while, at lower doses, it exhibits a bacteriostatic and hormetic effects [20]. The cell damage induced by NO may result in DNA mutations and strand breaks or nitrosylation of key proteins, including enzymes that may lose activity [21]. The ability of Mtb to survive and replicate within the MØs, even in the presence of adverse factors such as NO, seems essential for the development of the infection. The identification of mechanisms used by mycobacteria to evade nitrosative damage is an important goal since it could help to develop strategies for treating or preventing TB. For instance, there are Mtb genes known to be involved in resistance to the damage induced by nitric oxide [22]. The results reported in this study show that the structurally related TAT is to be considered a potential virulence factor of mycobacteria as well. TAT is highly recognized by serum antibodies in individuals with active tuberculosis [11], a fact that was once attributed to cross-reactivity of TAT to anti-DAT antibodies. More recently, structural studies of Mtb glycolipids have evidenced the presence of TAT in both clinical isolates and typical strains [23, 24]. To the best of our knowledge, this is the first report dealing with a biological activity of TAT on antimycobacterial immune response. Further studies will be needed to clarify whether the fatty acyl structural differences between species may account for the effects herein described.

## Conclusions

We show that DAT and TAT, two glycolipids located on the cell wall behave as virulence factors engaged in the downregulation of NO and iNOS, therefore representing a potential weapon of the mycobacteria against the innate immune response.

## Methods

### Ethics statement

Use of animals and experimental procedures were reviewed and approved by the Bioethics Committee of our Institute following established protocols.

### Isolation of glycolipids

*M. fortuitum* ATCC 6841 was used to isolate DAT and TAT, which are similar in *M. fortuitum* and Mtb. Non-covalently linked lipids were extracted from live bacilli using CHCI_3_/CH_3_OH (1:2, vol/vol) and CHCI_3_/CH_3_OH (2:1, vol/vol). Pooled extracts were dried and suspended in CHCl_3_/CH_3_OH/H_2_O (4:2:1, vol/vol/vol). After that, crude lipid extracts were dissolved in chloroform and applied to a Florisil column (Biotecna Corp., Miami, FL, USA). The elutions were performed with chloroform and methanol and fractionation of lipids was monitored by thin-layer chromatography (TLC) on silica gel-60 F_254_ coated plates (E. Merck, Darmstadt, Germany) developed with: CHCl_3_/CH_3_OH (9:1, vol/vol) as solvent I; CHCI_3_/CH_3_OH (8:2, vol/vol), as solvent II; or CHCI_3_/CH_3_OH/H_2_O (60:12:1, vol vol/vol), as solvent III. The sugar-containing compounds were visualized by spraying plates with 2 % anthrone in concentrated H_2_SO_4_ followed by heating at 110 °C. Acylated trehaloses appeared as anthrone-positive lipids (blue spots) with a Rf value of 0.37 for DAT and a Rf value of 0.64 - 0.68 for TAT. A final purification was carried in a TLC on silica gel-60 coated plates with a thickness of 0.5 mm (E. Merck, Darmstadt, Germany). The lipids were recovered from the plates and examined as mentioned before. The fractions with purified DAT were pooled, dried and subjected to the Lymulus test to verify endotoxin contamination. A similar procedure was followed to purify TAT.

### Characterization of dat and tat by fourier transform infrared spectroscopy

Fourier transform infrared spectroscopy (FTIR) analyses of the lipids were recorded in a Vector 33 FTIR spectrometer (Bruker Corporation, Billerica, MA, USA), equipped with an attenuated total reflection (ATR) module. About 0.5 mg of the product was dissolved in 200 μl chloroform-methanol (9:1, vol/vol) and placed into the ATR cell. FTIR spectrum measurement was performed in wave number range of 4000-450 cm^−1^.

### Assays to study the effects of dat and tat on nitric oxide production in macrophages

Six to seven week old Balb/c-J mice were used. To obtain MØs, bone marrow cells were flushed from femurs and tibias and cultured in RPMI 1640 with 20 % FBS, supplemented with 1 % non-essential aminoacids, 1 % of antibiotic-antimycotic and 1 % sodium pyruvate (Invitrogen, Eugene, OR, USA). Cells were grown at 37 °C with 5% CO_2_. At day 10, MØs were obtained, and the cell viability was assessed with Trypan blue. In preliminary experiments to determine the capacity of bone marrow-derived MØs from Balb/c-J mice to produce NO, the cells were treated with various amounts of LPS (E.coli B55:05; Sigma Chemical Co, St Louis, MO, USA) or recombinant IFN-γ (BioLegend, San Diego, CA, USA). The glycolipids were dissolved in hexane:ethanol (1:1, v/v). To each well, 20μg glycolipid in 100 μl hexane:ethanol were added and allowed to evaporate to dryness; control wells received solvent alone. Then, 1 × 10^6^ MØs were added to the wells and incubated for 24h at 37 °C with 5 % CO_2_. Afterward, 500 μg LPS or 250ng IFN-γ were added to the wells. Control wells with only hexane:ethanol, DAT or TAT were included. After 24 h the isolated supernatants were mixed with an equal volume of Griess reagent (1 % sulfanilamide, 0.1 % N-1-naphthylethylenediamine dihydrochloride, and 2 % phosphoric acid) (Promega Co., Madison,WI, USA) and incubated at room temperature for 10 min. Absorbance was measured at 550 nm.

### Western blot and flow cytometry to determine the expression of iNOS in macrophages treated with DAT and TAT

To investigate the expression of iNOS by MØs treated with the glycolipids, the cells were lysed with RIPA buffer and the proteins were separated in 7.5 % PAGE-SDS gels, transferred to a PVDF membranes and incubated overnight with a monoclonal antibody diluted 1:100 to murine iNOS (BD Biosciences, San Diego, CA, USA). Membranes were extensively rinsed with PBS and incubated with a secondary antibody to mice IgG diluted 1:200, 2 h at room temperature. The reactive bands were visualized by chemiluminescence with SuperSignal West Dura kit (Pierce, Rockford, IL, USA) or with DAB/H_2_O_2_. For flow cytometry, 5 × 10^5^ cells were fixed with paraformaldehyde 1 %, permeabilized with saponin 0.05 % and incubated 1 h with the monoclonal antibody diluted 1:200 to murine iNOS. The cells were rinsed and incubated for 1 h with a secondary antibody labeled with FITC. The cells were analyzed in a Beckton Dickinson cytofluorometer (San Diego, CA, USA). As a control, a monoclonal antibody of the same isotype was used.

### Statistical analysis

Statistical analysis was performed using the standard statistical software Prism version 5.0, GraphPad Software, (San Diego, CA, USA). NO and iNOS production by cells was expressed as inhibition percentages, where LPS or IFN-γ induced levels, in the absence of mycobacterial lipids, were taken as 100 %.
